# Endoplasmic reticulum stress mediates parathyroid hormone-induced apoptosis in vascular smooth muscle cells

**DOI:** 10.1080/0886022X.2022.2027248

**Published:** 2022-02-16

**Authors:** Shuzhong Duan, Xinpan Chen, Yingjie Liu, Weikang Guo, Wenhu Liu

**Affiliations:** a Department of Nephrology, Beijing Friendship Hospital, Faculty of Kidney Diseases, Capital Medical University, Beijing, China; b Department of Nephrology, Chengde Medical University Affiliated Hospital, Chengde, China

**Keywords:** CKD, vascular calcification, PTH, ER stress, apoptosis

## Abstract

Vascular calcification is one of the most common complications of chronic kidney disease (CKD), which is closely associated with increased mortality and morbidity rates of CKD patients. It has been reported that increased parathyroid hormone (PTH) aggravates vascular calcification in CKD patients. However, the direct role of PTH in vascular smooth muscle cells (VSMCs) is less elucidated. Here, we present evidence that PTH promotes apoptosis of VSMCs and endoplasmic reticulum (ER) stress participates in this process. Human aorta vascular smooth muscle cells (HASMCs) were treated with different concentrations of PTH for various time. HASMC apoptosis was detected by flow cytometry. Expression of phosphorylated (p)-PERK, CHOP, IRE1, p-JNK, and cleaved caspase 3 was determined by Western blotting. We found that PTH induced HASMC apoptosis and increased the expression of cleaved caspase 3. Furthermore, PTH activated PERK-CHOP and IRE1-JNK ER stress pathways. Either inhibition of JNK by SP600125 or CHOP by siRNA ameliorated PTH-induced apoptosis in HASMCs. We therefore suggest that ER stress participates in PTH-induced apoptosis of VSMCs, which may be a possible mechanism of PTH-promoted vascular calcification in CKD patients.

## Introduction

Vascular calcification is highly prevalent in chronic kidney disease (CKD) patients. The presence of vascular calcification in CKD is associated with major adverse cardiovascular events [1]. Vascular smooth muscle cells (VSMCs) play an integral role in mediating vascular calcification of CKD. A large amount of evidence supports that VSMC apoptosis is a major mechanism of vascular calcification in CKD [2]. Many studies have indicated the involvement of endoplasmic reticulum (ER) stress-induced VSMC apoptosis in vascular calcification [3–6].

The ER is an essential organelle that participates in protein quality control of all eukaryotic cells. ER homeostasis is critical to control various intracellular physiological functions including protein folding, protein translocation, lipid metabolism, cell differentiation, and calcium homeostasis [7]. There are three main branches of ER stress: inositol-requiring enzyme 1α (IRE1α), PRK-like ER kinase (PERK), and activating transcription factor (ATF) 6. When the ER is operating under homeostatic conditions, IRE1α, PERK, and ATF6 are kept in monomeric and inactive states through interactions with an ER chaperone called 78-kDa glucose-regulated protein/immunoglobulin-binding protein (GRP78/BiP). However, under many pathological conditions, misfolded proteins accumulate in the ER to an unmanageable level, which activates IRE1α, PERK, or ATF6 to induce ER stress. When the pathological condition is short and reversible, ER stress can save the cell fate. However, if cellular damage persists and induces chronic ER stress, cells trigger the apoptosis pathway. ER stress-induced apoptosis is mainly mediated through PERK and IRE1 pathways. PERK activates a prosurvival mechanism, but switches to a proapoptotic mechanism under prolonged ER stress by regulating ATF4 and CAAT/enhancer-binding protein homologous protein (CHOP). IRE1 activates the c-Jun N-terminal kinase (JNK) pathway that ultimately triggers apoptosis [8,9]. Capase-12 localized to the ER and activated by ER stress has been identified to mediate ER stress-induced apoptosis [10,11].

In CKD patients, a high level of parathyroid hormone (PTH) triggers vascular calcification that increases mortality [12,13]. A high PTH level is also a predictor of the progression of coronary artery calcification in patients on dialysis [14]. Therefore, it is important to elucidate how PTH regulates vascular calcification. Our previous study showed that PTH induces VSMC apoptosis [15], which may contribute to VSMC calcification, but the mechanism of PTH-induced apoptosis remained unknown. In this study, we found that PERK and IRE1-mediated ER stress pathways participate in PTH-induced VSMC apoptosis.

## Materials and methods

### Cell culture

Primary human aortic smooth muscle cells (HASMCs) were purchased from ScienCell Research Laboratories (Carlsbad, USA) and cultured as described previously [16]. Briefly, HASMCs were cultured at 37 °C in a humidified atmosphere with 5% CO_2_ in smooth muscle cell growth culture medium (1% smooth muscle cell growth supplement in basal medium with 2% FBS and 1% penicillin-streptomycin (ScienCell)). The medium was replaced every other day. All experiments used third to fifth passage cells. Human PTH fragment 1–34 was purchased from Sigma-Aldrich (P3796). HASMCs were treated with various concentrations (1 × 10^−6^, 1 × 10^−7^, or 1 × 10^−8 ^mol/L) of PTH for different time (0, 3, 7, 10, or 14 days), HASMCs cultured in normal medium for the same days as control. Smooth muscle cell growth culture medium was used for all experiments.

### Transfection of short interfering RNA (siRNA)

SiRNAs against human CHOP and a control siRNA were purchased from YMBio (Beijing, China). The siRNA design principles were based on Tuschl rules and the siRNAs were designed using GenePharma Rnai Designer V2.0 software. The gene sequences were as follows: CHOP sense: 5′-GAG CUC UGA UUG ACC GAA UTT-3′ and antisense: 5′-AUU CGG UCA AUC AGA GCU CTT-3′); control scrambled siRNA sense: 5′-UUC UCC GAA CGU GUC ACG UTT-3′ and antisense: 5′-ACG UGA CAC GUU CGG AGA ATT-3′. SiRNAs were transfected into cells using a Lipofectamine™ 2000 Reagent kit (Invitrogen) as described previously [17]. Briefly, cells were seeded on a six-well plate in culture medium without antibiotics. At 30%–50% confluence, the cells were used for transfection. Opti-MEM I (Invitrogen) was used to dilute the siRNA. Lipofectamine™ 2000 Reagent was added to the mixture of siRNAs, then cells were incubated with the mixture for 20 min at room temperature. The cells were then incubated with the RNAi duplex-Lipofectamine™ 2000 complexes without serum for 6 h at 37 °C. Finally, the medium was replaced and cells were incubated for another 48 h.

### Flow cytometry

An Annexin V-FITC/PI Apoptosis Detection Kit was purchased from KeyGEN BioTECH (KGA108). An Annexin V-APC/7-AAD Apoptosis Detection Kit was purchased from SUNGEN BIOTECH (AO2001-11A). Cells were seeded on six-well plates at a density of 2 × 10^5^ cells/well. When the stimulus time point was reached, PBS was used to wash the cells and trypsin was applied to harvest the cells. Then, 100 μL binding buffer was added to the cells and 5 μL Annexin V-FITC and 5 μL propidium iodide (PI) were added, followed by mixing. Then, the cells were maintained in a darkroom at room temperature for 15 min, followed by addition of another 400 μL binding buffer. Double-stained cells in each well were subsequently analyzed by a FACSCanto flow cytometer (BD, USA). At least 10 000 cells were counted in each analysis. We used the annexin V-FITC/PI apoptosis detection method to assess PTH-induced HASMC apoptosis. Annexin V is used in flow cytometry to identify cells undergoing apoptosis based on its ability to bind to phosphatidylserine (PS). Early apoptotic cells (PS+/PI−), late apoptotic cells (PS+/PI+), and viable cells (PS−/PI−) were identified by flow cytometry. Apoptotic cells were calculated by the sum of early and late apoptotic cells. The experiments were repeated at least three times.

### Western blot analysis

Total proteins were extracted from cells with RIPA lysis buffer. The concentration of proteins was measured by a bicinchoninic acid protein assay kit (Thermo Fisher Scientific, Waltham, MA, USA). Each sample was combined with an equal volume of SDS loading buffer and sonicated for 10 s. The proteins were heated at 95 °C for 5 min and then placed on ice for 5 min. Subsequently, the samples were resolved on 8%–12% SDS-PAGE gels (Solarbio). Then, the proteins were electrophoretically transferred onto nitrocellulose membranes (Amersham International, Cardiff, UK). For blocking, 5% dry nonfat milk was applied to the membranes for 2 h at room temperature. Then, the membranes were incubated with primary antibodies overnight at 4 °C. After washing in TBST, horseradish peroxidase-conjugated secondary antibodies were applied to the membranes at room temperature for 1 h. The membranes were washed with TBST and developed using an enhanced chemiluminescence kit (Millipore Co., Bedford, MA, USA). Then, the membranes were exposed to a Kodak X-OMAT film (Eastman Kodak, Rochester, NY, USA). The primary antibodies and dilutions were as follows: anti-cleaved caspase 3 (1:1000, Cell Signaling Technology, #9662), anti-caspase 3 (1:1000, Cell Signaling Technology, #9662), anti-IRE1 (1:1000, Abcam, ab37073), anti-p-PERK (1:1000, Thermo Fisher Scientific, PA5-40294), anti-PERK (1:1000, Cell Signaling Technology, #3192), anti-p-JNK (1:500, Cell Signaling Technology, #9255), anti-JNK (1:500, Proteintech, 51151-1-AP), anti-CHOP (1:1000, Proteintech, 15204-1-AP), anti-caspase 12 (1:1000, Abcam, ab62484), anti-GRP78/BiP (1:1,000, Proteintech, 11587-1-AP), anti-p-eIF2 (1:1000, Affinity, AF3087), anti-eIF2 (1:1000, Affinity, AF6087), anti-ATF4 (1:1000, Affinity, DF6008), and anti-GAPDH (1:3000, Cell Signaling Technology, #5174).

### Real-time PCR

Total RNA was extracted using Trizol reagent (Invitrogen). The concentration was quantified by UV-Vis Spectrophotometry (NanoDrop Technologies). A high-capacity cDNA reverse transcription kit (Applied Biosystems) was used for reverse transcription. Quantitative real-time reverse transcription-PCR (RT-PCR) was carried out using a 7300 real-time PCR System (Applied Biosystems, CA, USA) with Power SYBR Green PCR Master Mix (Applied Biosystems) All cDNA samples were analyzed in triplicate. The gene expression analysis was performed using the comparative threshold cycle (2-ΔΔCT) method. Glyceraldehyde 3-phosphate dehydrogenase (GAPDH) was used as an internal control. The following primer sequences were used: CHOP forward primer 5′-AAT CTT CAC TCT TGA CCC T-3′ and reverse primer 5′-ATG ACC ACT CTG TTT CCG TTT C-3′; Bax forward primer 5′-AAG CTG AGC GAG TGT CT-3′ and reverse primer 5′-GTT CTG ATC AGT TCC GGC AC-3′; Bad forward primer 5′-GAG GAC GAA GGG ATG G-3′ and reverse primer 5′-AAG TTC CGA TCC CAC CAG G-3′; Bim forward primer 5′-GGC AAA GCA ACC TTC TGA TG-3′ and reverse primer 5′-TGT CTG TAG GGA GGT AGG GG-3′; Bcl-2 forward primer 5′-GCC TTC TTT GAG TTC GGT GG-3′ and reverse primer 5′-GAA ATC AAA CAG AGG CCG CA-3′; GAPDH forward primer 5′-AGA AGG CTG GGG CTC ATT TG-3′ and reverse primer 5′-AGG GGC CAT CCA CAG TCT TC-3′.

### Statistical analysis

Data were presented for at least three independent experiments. GraphPad Software was used for statistical analysis of all independent experiments. The results are shown as the mean ± SD. The statistical significance of differences between two groups was calculated by one-way ANOVA. A *p*-value of less than .05 indicated significance (*p < .05).

## Results

### PTH promotes HASMC apoptosis

**Figure 1.**
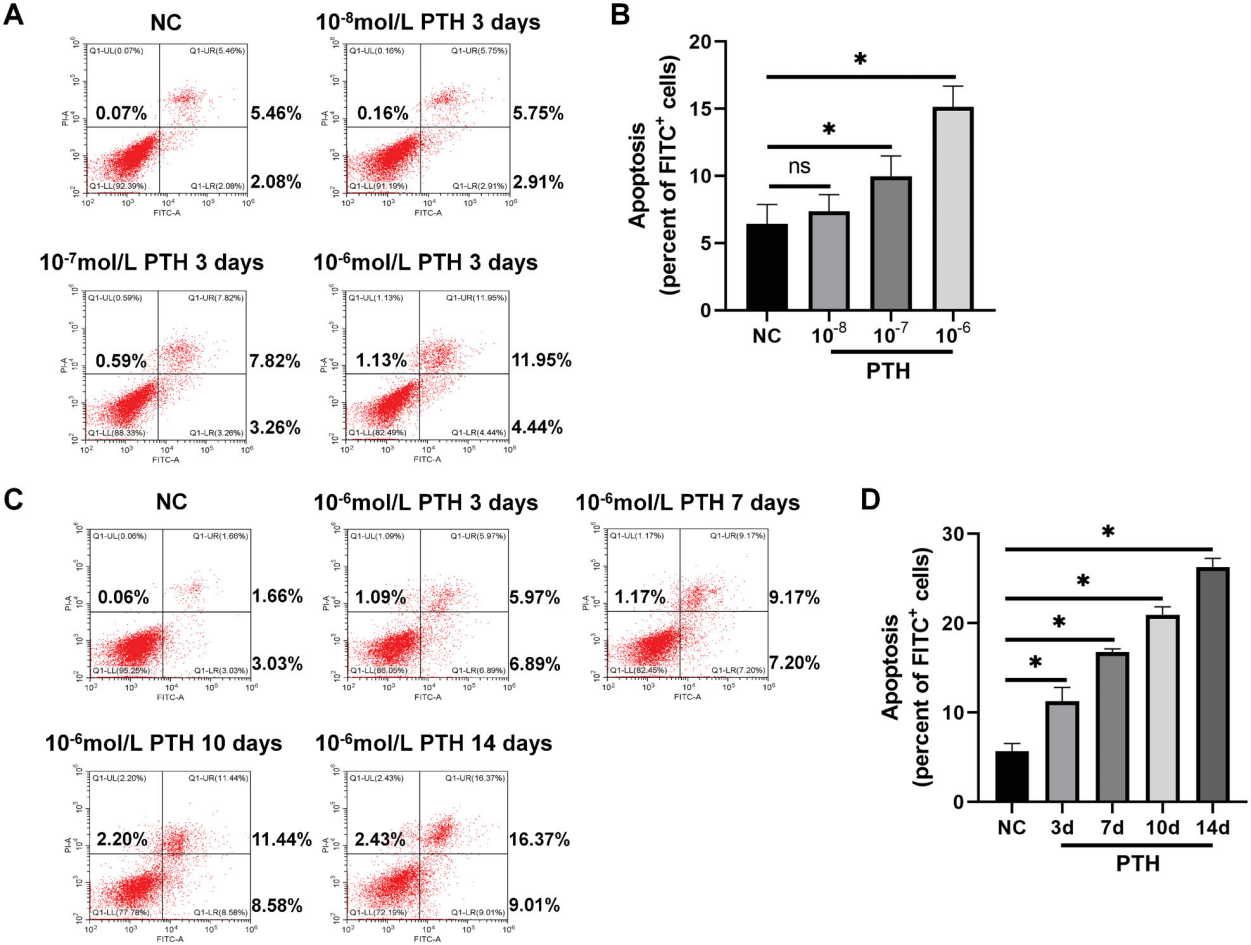
PTH induces HASMC apoptosis. Apoptosis was detected by flow cytometry. (A) HASMCs were treated with 1 × 10^−8^–1 × 10^−6 ^mol/L PTH for three days. (B) Data are the mean ± SD of three independent experiments. (C) HASMCs were treated with 1 × 10^−6 ^mol/L PTH for 0–14 days. Percentages of PS-positive/PI-negative and PS-positive/PI-positive cells are shown. (D) Data are the mean ± SD of three independent experiments. HASMCs cultured in normal medium for the same days as control. *p < .05 versus control.

**Figure 2.**
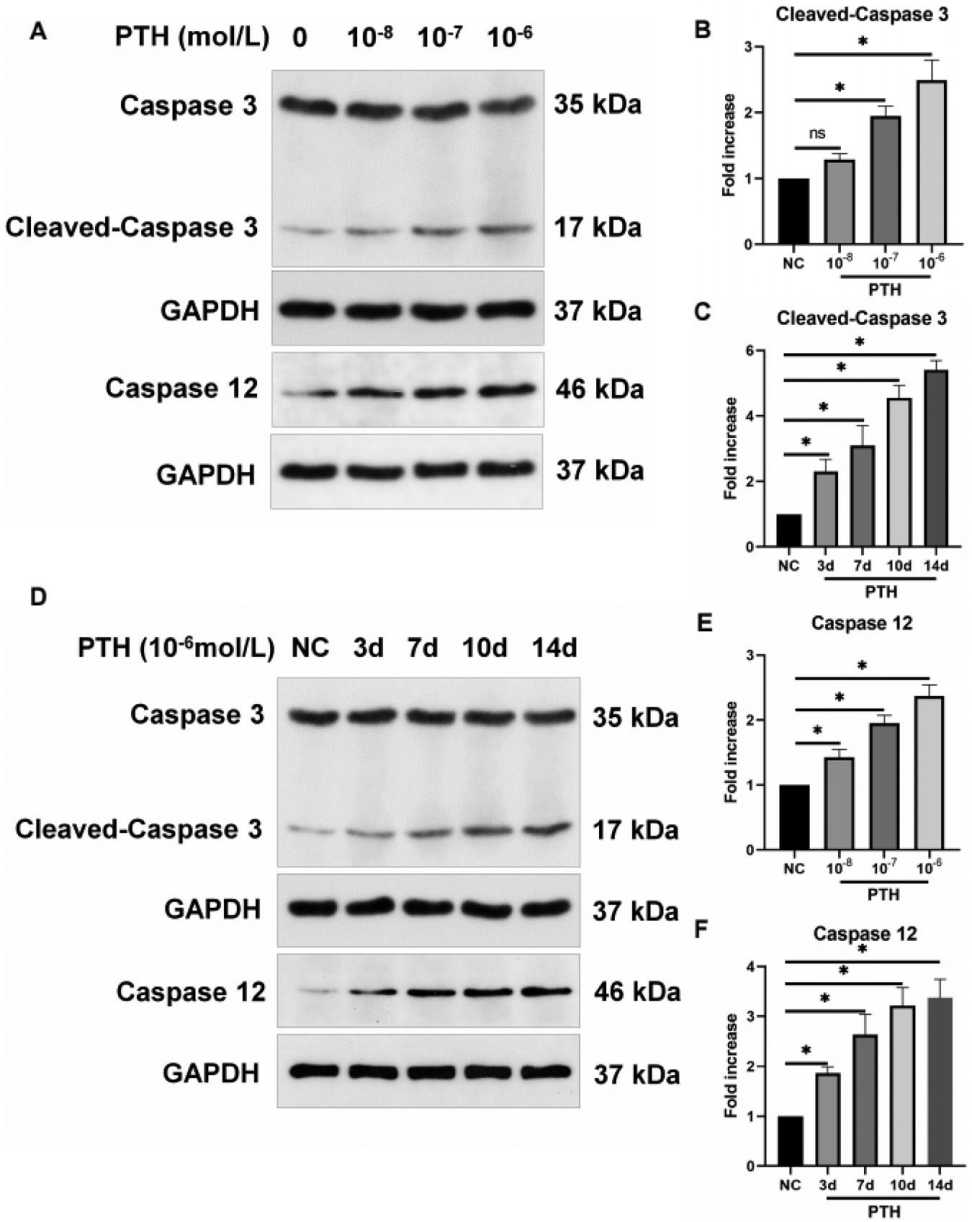
PTH increases cleaved caspase 3 and caspase 12 expression as detected by Western blot analysis. (A) HASMCs were treated with 1 × 10^−8^–1 × 10^−6 ^mol/L PTH for three days. (B, E) Data are the mean ± SD of three independent experiments. (D) HASMCs were treated with 1 × 10^−6 ^mol/L PTH for 0–14 days. (C, F) Data are the mean ± SD of three independent experiments. HASMCs cultured in normal medium for the same days as control. *p < .05 versus control.

**Figure 3.**
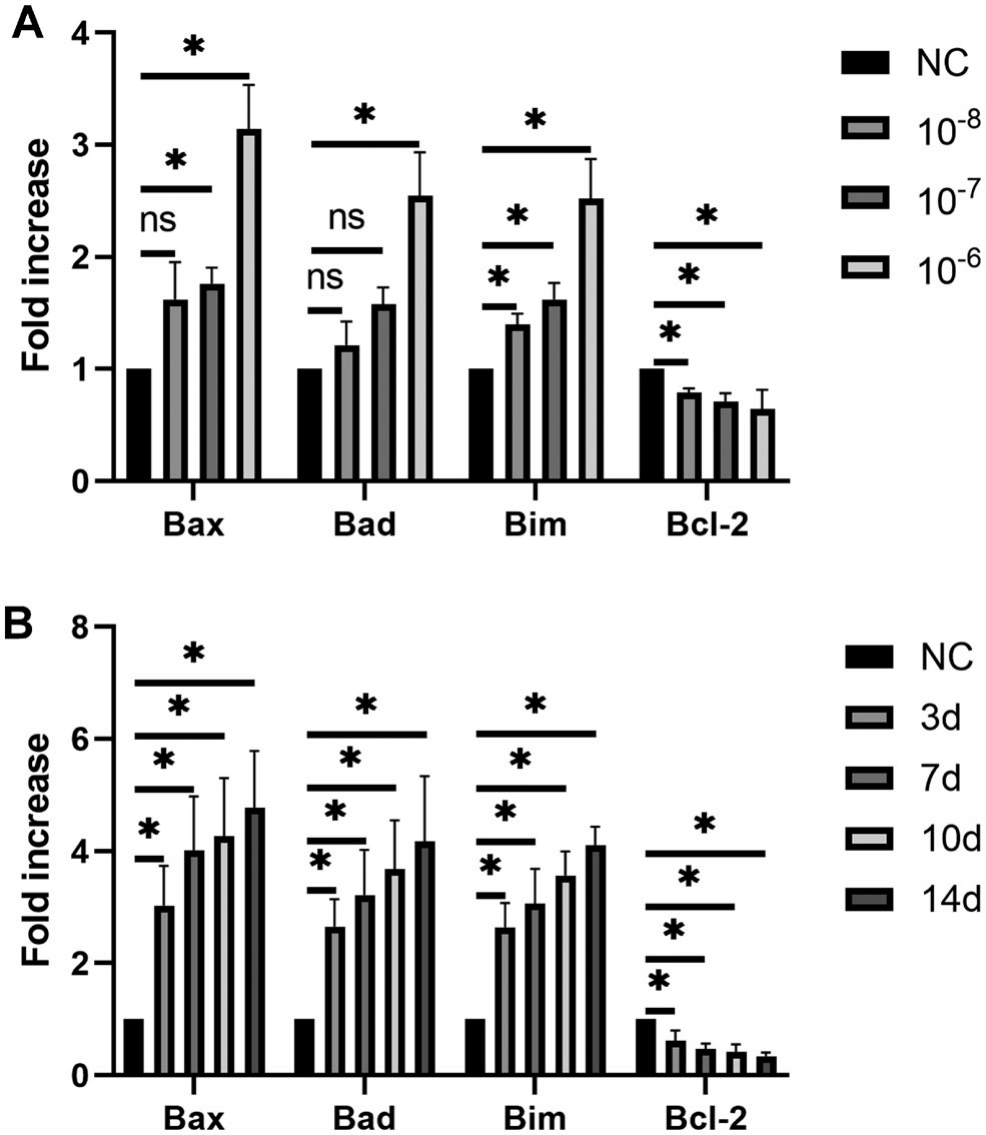
PTH increases Bax, Bad, and Bim expression and decreases Bcl-2 expression as detected by RT-PCR. (A) HASMCs were treated with 1 × 10^−8^–1 × 10^−6 ^mol/L PTH for three days.(B) HASMCs were treated with 1 × 10^−6 ^mol/L PTH for 0–14 days. HASMCs cultured in normal medium for the same days as control. *p < .05 versus control.

HASMCs were treated with various concentrations of PTH for three days, which induced apoptosis in a dose-dependent manner (Figure 1(A,B)). At 1 × 10^−6 ^mol/L, HASMC apoptosis induced by PTH was the most severe. Next, we used 1 × 10^−6 ^mol/L PTH to treat HASMCs for 0, 3, 7, 10, and 14 days. The results showed that, as the stimulation time increased, the number of apoptotic cells was also increased (Figure 1(C,D)). Furthermore, we analyzed cleaved caspase 3, a critical effector of apoptosis. We found that PTH increased cleaved caspase 3 expression in dose- and time-dependent manners (Figure 2A–D). Next, we measured the expression of caspase 12, a marker of ER stress-induced apoptosis [18,19]. We found that PTH also increased caspase 12 expression in dose- and time-dependent manners (Figure 2(A, D, E, F)). We also used RT-PCR to detect the expression of proapoptotic markers (Bax, Bad, and Bim) and an anti-apoptotic marker (Bcl-2). We found that PTH increased Bax, Bad, and Bim expression and decreased Bcl-2 expression (Figure 3(A,B)). HASMC apoptosis is the major mechanism of vascular calcification in CKD [20]. Therefore, we assumed that PTH may accelerate vascular calcification by inducing HASMC apoptosis.

### PTH induces ER stress in HASMCs

**Figure 4.**
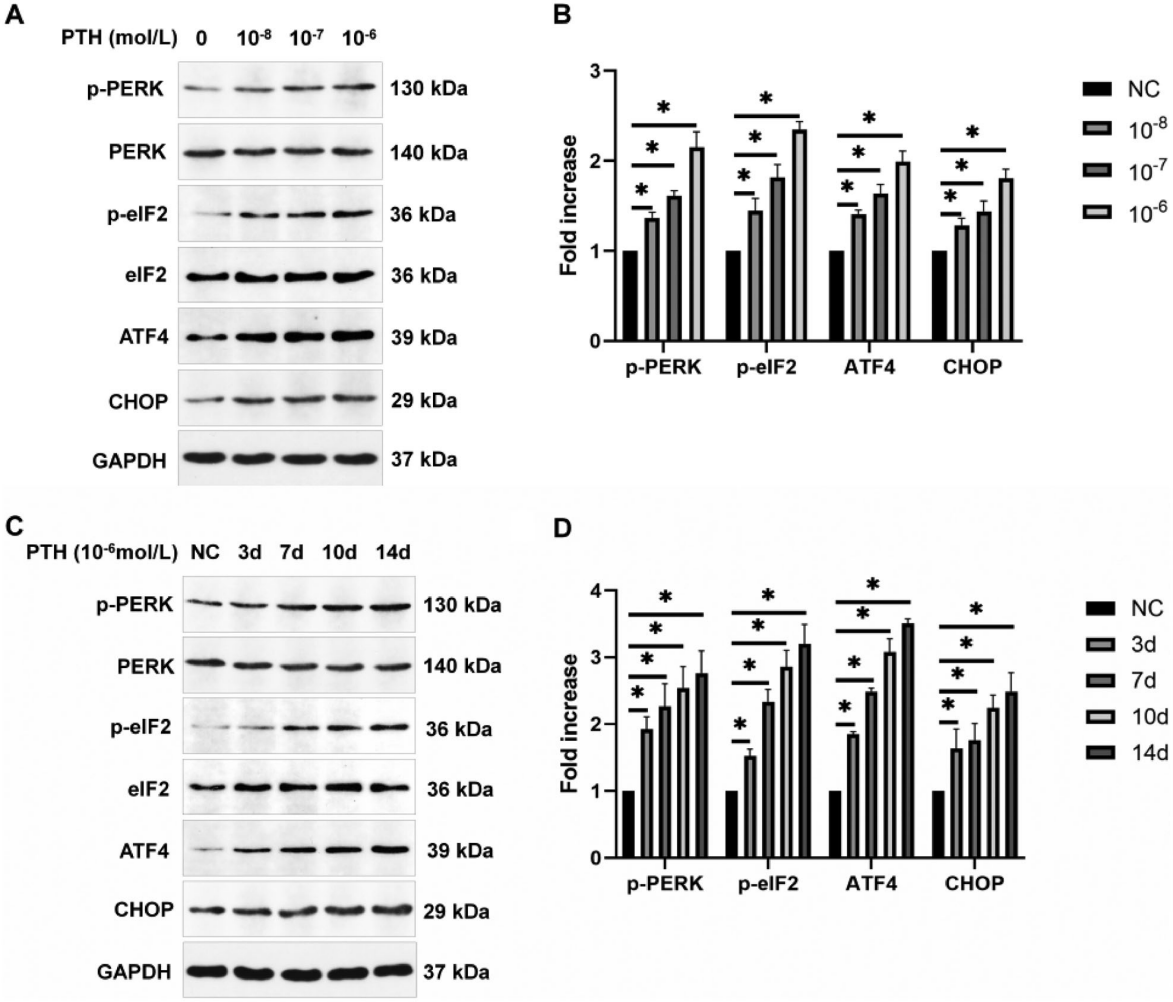
PTH activates the PERK-CHOP ER stress pathway as detected by Western blot analysis. (A) HASMCs were treated with 1 × 10^−8^–1 × 10^−6 ^mol/L PTH for three days. (B) Data are the mean ± SD of three independent experiments. (C) HASMCs were treated with 1 × 10^−6 ^mol/L PTH for 0–14 days. (D) Data are the mean ± SD of three independent experiments. HASMCs cultured in normal medium for the same days as control. *p < .05 versus control.

**Figure 5.**
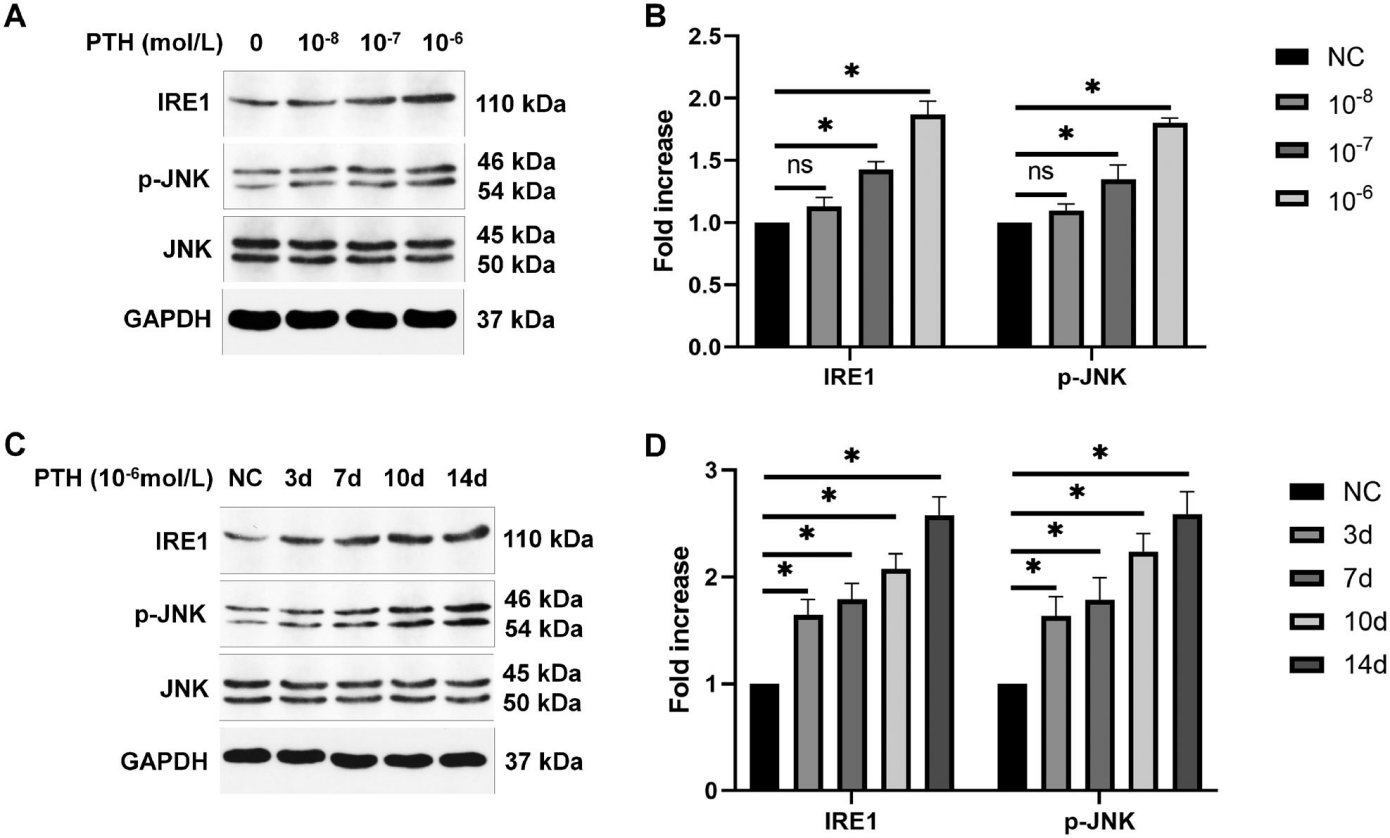
PTH activates the IRE1-JNK ER stress pathway as detected by Western blot analysis. (A) HASMCs were treated with 1 × 10^−8^–1 × 10^−6 ^mol/L PTH for three days. (B) Data are the mean ± SD of three independent experiments. (C) HASMCs were treated with 1 × 10^−6 ^mol/L PTH for 0–14 days. (D) Data are the mean ± SD of three independent experiments. HASMCs cultured in normal medium for the same days as control. *p < .05 versus control.

**Figure 6.**
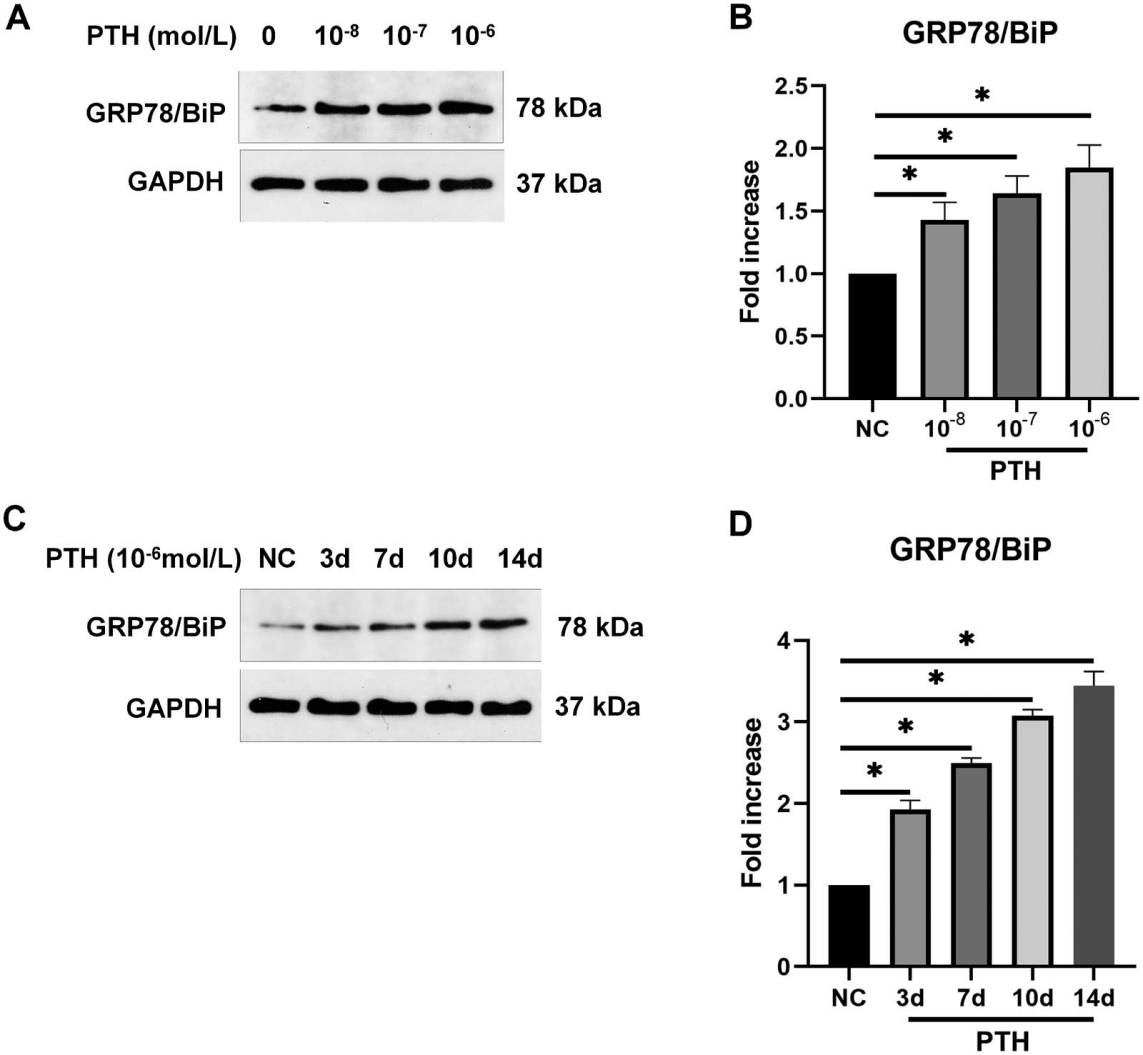
PTH increases GRP78/BiP expression as detected by Western blot analysis. (A) HASMCs were treated with 1 × 10^−8^–1 × 10^−6 ^mol/L PTH for three days. (B) Data are the mean ± SD of three independent experiments. (C) HASMCs were treated with 1 × 10^−6 ^mol/L PTH for 0–14 days. (D) Data are the mean ± SD of three independent experiments. HASMCs cultured in normal medium for the same days as control. *p < .05 versus control.

Sustained ER stress can lead to apoptosis. PERK-eIF2α-ATF4-CHOP and IRE1-JNK are the main pathways of ER stress-induced apoptosis. Therefore, we detected the related protein expression by Western blotting. We found that the expression of p-PERK, p-eIF2α, ATF4, and CHOP was increased gradually after treating HASMCs with various doses of PTH for 3 days (Figure 4(A,B)). Next, we measured the expression of p-PERK/p-eIF2α/ATF4/CHOP after treating HASMCs with 1 × 10^−6 ^mol/L PTH for 0–14 days. We found that the expression of p-PERK/p-eIF2α/ATF4/CHOP was increased over time (Figure 4(C,D)). Next, we detected the protein expression of IRE1 and p-JNK. We found that PTH also increased the expression of IRE1 and p-JNK in dose- and time-dependent manners (Figure 5(A,D)). GRP78/BiP is a central regulator of ER functions because of its roles in protein folding and assembly as well as controlling the activation of transmembrane ER stress sensors. Therefore, we detected GRP78/BiP expression by Western blotting. We found that the expression of GRP78/BiP was also increased by PTH (Figure 6(A,D)). These results suggested that PTH induced HASMC apoptosis *via* PERK-CHOP and IRE1-JNK ER stress pathways.

### PTH induces HASMC apoptosis *via* PERK-CHOP and IRE1-JNK ER stress pathways

**Figure 7.**
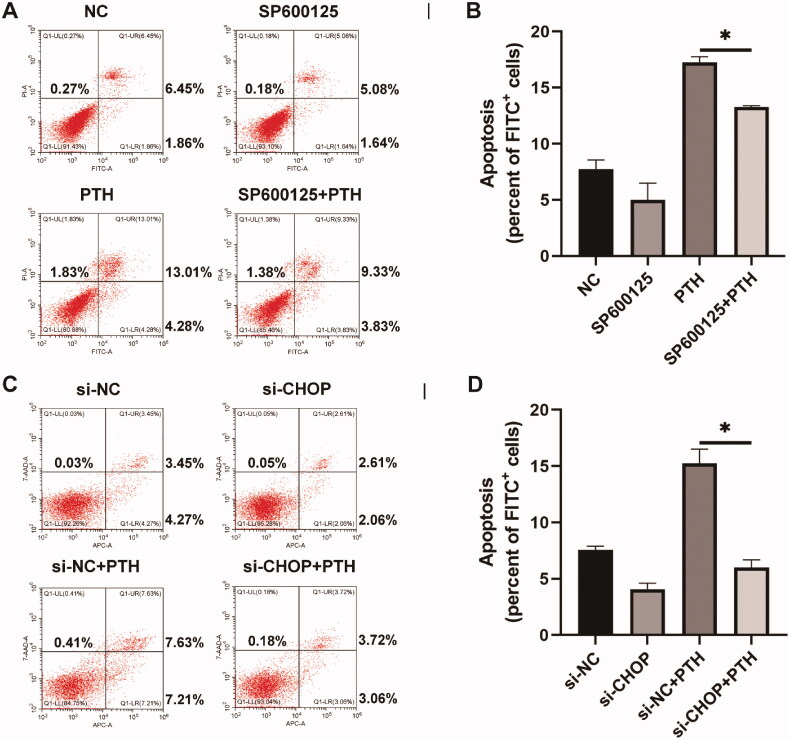
Inhibition of JNK or CHOP blocks PTH-induced HASMC apoptosis. Apoptosis was detected by flow cytometry. (A) HASMCs were pretreated with SP6000125, a JNK inhibitor, and then treated with 1 × 10^−6 ^mol/L PTH for 3 days. (B) Data are the mean ± SD of three independent experiments. (C) HASMCs were transfected with si-CHOP and then treated with 1 × 10^−6 ^mol/L PTH for three days. (D) Data are the mean ± SD of three independent experiments. HASMCs cultured in normal medium for the same days as control. *p < .05 versus PTH group.

**Figure 8.**
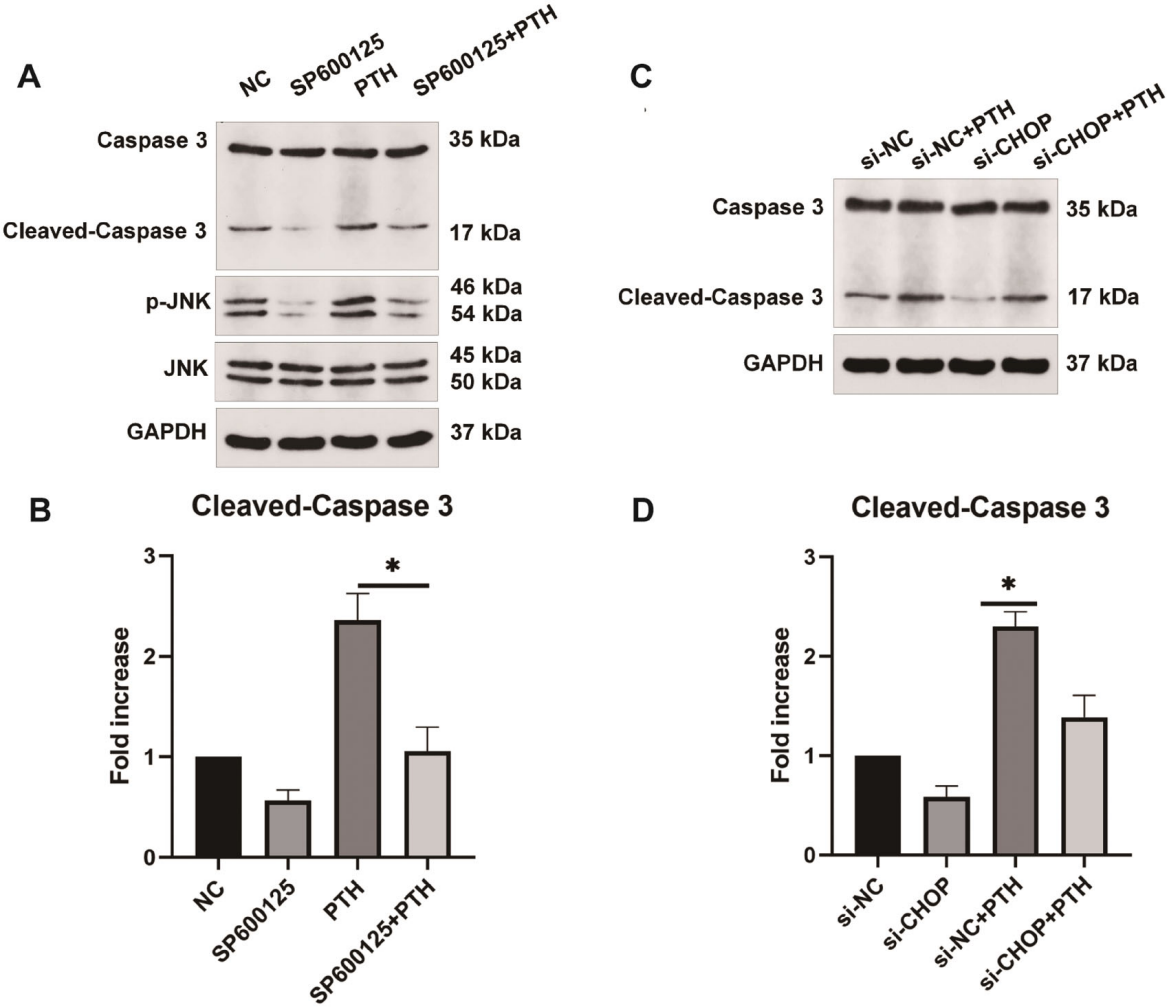
Inhibition of JNK or CHOP suppresses PTH-induced cleaved caspase 3 expression as detected by Western blot analysis. (A) HASMCs were pretreated with SP6000125, a JNK inhibitor, and then treated with 1 × 10^−6 ^mol/L PTH for three days. HASMCs cultured in normal medium for the same days as control. (B) Data are the mean ± SD of three independent experiments. (C) HASMCs were transfected with si-CHOP and then treated with 1 × 10^−6 ^mol/L PTH for three days. HASMCs cultured in scramble siRNA for the same days as control. (D) Data are the mean ± SD of three independent experiments. *p < .05 versus PTH group.

To verify that PTH induced HASMC apoptosis *via* PERK-CHOP and IRE1-JNK ER stress pathways, we used SP600125, a JNK-specific inhibitor, to pretreat HASMCs for 24 h and then stimulated the cells with 1 × 10^−6 ^mol/L PTH for three days. We used the annexin V-FITC/PI or annexin V-APC/7-AAD apoptosis detection methods to assess PTH-induced HASMC apoptosis. We found that inhibition of JNK suppressed PTH-induced HASMC apoptosis (Figure 7(A,B)). Furthermore, we applied siRNA against CHOP (siCHOP) before treating HASMCs with PTH. The result of the transfection effect is shown in the supplementary figure. Apoptosis was decreased after transfection of siCHOP (Figure 7(C,D)). We found that suppression of CHOP decreased PTH-induced HASMC apoptosis. Furthermore, we used SP600125 or siCHOP to treat HASMCs before stimulation with PTH, then detected the expression of cleaved caspase 3. We found that both SP600125 and siCHOP inhibited PTH-induced cleaved caspase 3 expression (Figure 8(A–D)). These results indicated that PERK-CHOP and IRE1-JNK ER stress pathways participated in PTH-induced HASMC apoptosis.

## Discussion

Secondary hyperparathyroidism and high serum phosphate are strong precipitating factors of vascular calcification, which are associated with poor survival. However, there is little evidence regarding a direct mechanism of PTH in vascular calcification. It is believed that HASMC apoptosis is a major mechanism of vascular calcification in CKD. In this study, we demonstrated that PTH promoted HASMC apoptosis. This may be one mechanism, whereby PTH promotes the development of vascular calcification in CKD patients.

Cardiovascular events are the main cause of death from CKD. Vascular calcification is prevalent in CKD patients and the main pathogenesis of cardiovascular events [21]. PTH is the major factor that promotes vascular calcification in CKD [22]. It has been reported that high serum phosphate and PTH distinctly regulate bone loss and vascular calcification in experimental CKD rats [13]. However, the underlying mechanism remained to be explored. The main mechanisms of vascular calcification in CKD include apoptosis, osteoblast transdifferentiation, extracellular vesicle secretion, dysregulation of procalcification/inhibitory factors, matrix remodeling, autophagy, inflammation, and cellular senescence [23]. Recently, we found that PTH induced HASMC apoptosis by upregulating sirtuin 1 [15], which indicated new target to prevent PTH-induced vascular calcification. In this study, we observed that PTH induced HASMC apoptosis in dose- and time-dependent manners. Additionally, PTH increased the expression of cleaved caspase 3, an apoptosis inducer [24].

Recent studies have suggested roles of the ER stress-mediated apoptosis pathway in some types of cardiovascular disorders, especially vascular calcification [25–32]. The ER is the organelle of synthesis, folding, and modification of secretory and cell surface proteins. ER dysfunction causes aberrant protein folding in the ER lumen. The accumulation of aberrant unfolded proteins induces ER stress that upregulates the capacity of the ER to process abnormal proteins. ER stress initially promotes cell survival; however, if endoplasmic reticulum stress continues or prolongs, it will activate the pathway leading to cell death. There are three transmembrane ER stress sensors, IRE1, PERK, and ATF6. They transduce information regarding the ER protein-folding status to the nucleus *via* the cytosol to reestablish the protein-folding capacity. When unfolded proteins accumulate in the ER, the sensors activate and increase the processing capacity for unfolded proteins. However, if the situation is beyond the ER capacity, IRE1 and PERK conduct apoptotic signals through CHOP or JNK and promote apoptosis to remove injured cells. We determined whether PTH induced HAMSC apoptosis *via* ER stress. We found that PTH stimulated both PERK-CHOP and IRE1-JNK ER stress apoptotic pathways. Next, we used siRNA that targeted CHOP to block the PERK-CHOP pathway and found that siCHOP suppressed PTH-induced apoptosis. Additionally, JNK-specific inhibitor SP600125 suppressed PTH-induced apoptosis. These results further showed that PERK-CHOP and IRE1-JNK ER stress pathways participated in PTH-induced apoptosis.

Our study also has some limitations. In accordance with the 2017 KDIGO CKD-Mineral and Bone Disorder guideline, in patients with CKD-5D, a target PTH range of two to nine times the upper limit of normal is recommended. In our center, the normal value of PTH is 11–62 pg/ml (1.2–6.5 × 10^−12 ^mol/L) and PTH in CKD-5D patients is usually maintained at 100–600 pg/ml (1.1–6.3 × 10^−11 ^mol/L). After long-term accumulation, PTH causes numerous pathophysiological changes that include vascular calcification. When we examined the mechanisms of PTH *in vitro*, much larger doses than pathological concentrations in patients were required. PTH contains 84 amino acids, and the active N-terminal fragment of PTH (residues 1–34) represents the first 34 amino acids of the mature hormone, which reproduces all activity of the full-length mature hormone. PTH 1–34 actives both parathyroid 1 and 2 receptors. Therefore, PTH 1–34 is often used to study the mechanisms of PTH. *In vitro* studies, 1 × 10^−6^–1 × 10^−10 ^mol/L PTH 1–34 is used to treat cells for 1–14 days for different objectives such as vascular calcification and bone osteogenesis [13,33–37]. The mostly used dose is 1 × 10^−6^–1 × 10^−8 ^mol/L. Therefore, in our study, we also used 1 × 10^−6^–1 × 10^−8 ^mol/L PTH 1–34 to treat VSMCs. Additionally, to better analyze the effect of PTH on VSMC apoptosis, we used five time points of 0, 3, 7, 10, and 14 days. Although *in vitro*, we demonstrated that PTH induced VSMC apoptosis and that this process was mediated by ER stress. However, it is important to verify whether ER stress-mediated apoptosis is an essential mechanism in vascular calcification of CKD *in vivo*, which is our next study. ATF6 is also an important ER stress sensor. It mediates mechanisms other than apoptosis and induces osteogenic differentiation by interacting with Runx2 [38]. Therefore, whether PTH activates ATF6 remains to be verified. The endoplasmic reticulum is the most important Ca^2+^ store in cells. When intracellular Ca^2+^ is disrupted, it causes ER stress. Our study showed that PTH activated ER stress, but how PTH activates ER stress and whether it is caused by disrupting the internal environment of Ca^2+^ in the endoplasmic reticulum remain to be verified by further experiments.

Taken together, we found that PTH induced HAMSC apoptosis, and PERK-CHOP and IRE1-JNK ER stress pathways participated in PTH-induced apoptosis *in vitro*. Thus, identification of the mechanism underlying how PTH injures VSMCs may provide new information and hypotheses regarding the regulation of vascular calcification in CKD.

## Supplementary Material

Supplemental Material

Supplemental Material
