# Supplementary figures and images for: Endoplasmic reticulum stress mediates parathyroid hormone-induced apoptosis in vascular smooth muscle cells
Source: Ren Fail. 2022 Feb 16;44(1):126–36. doi: 10.1080/0886022X.2022.2027248 (PMC8856047; doi:10.1080/0886022X.2022.2027248)

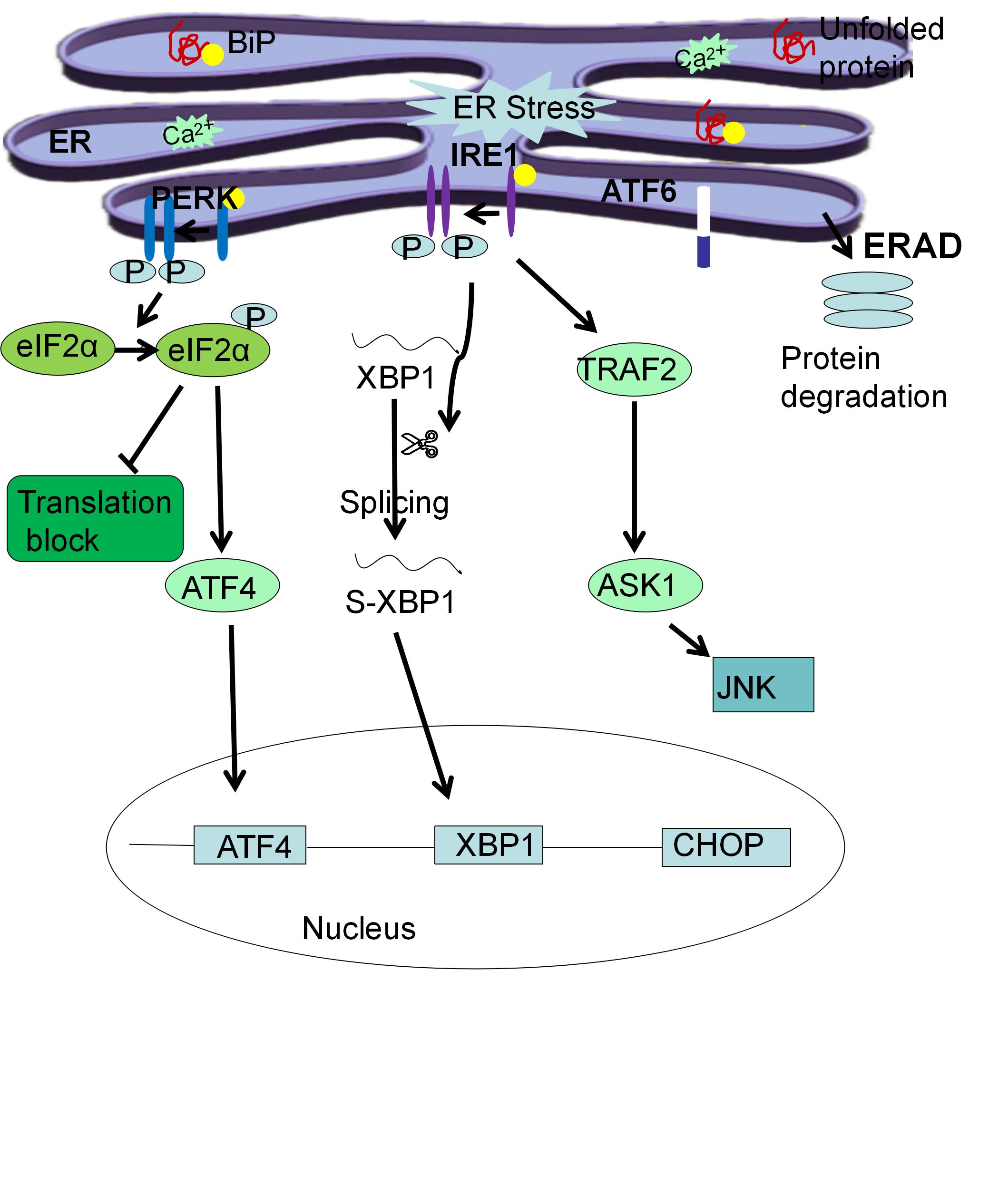

Supplement: Supplemental Material [file IRNF_A_2027248_SM9110.png]

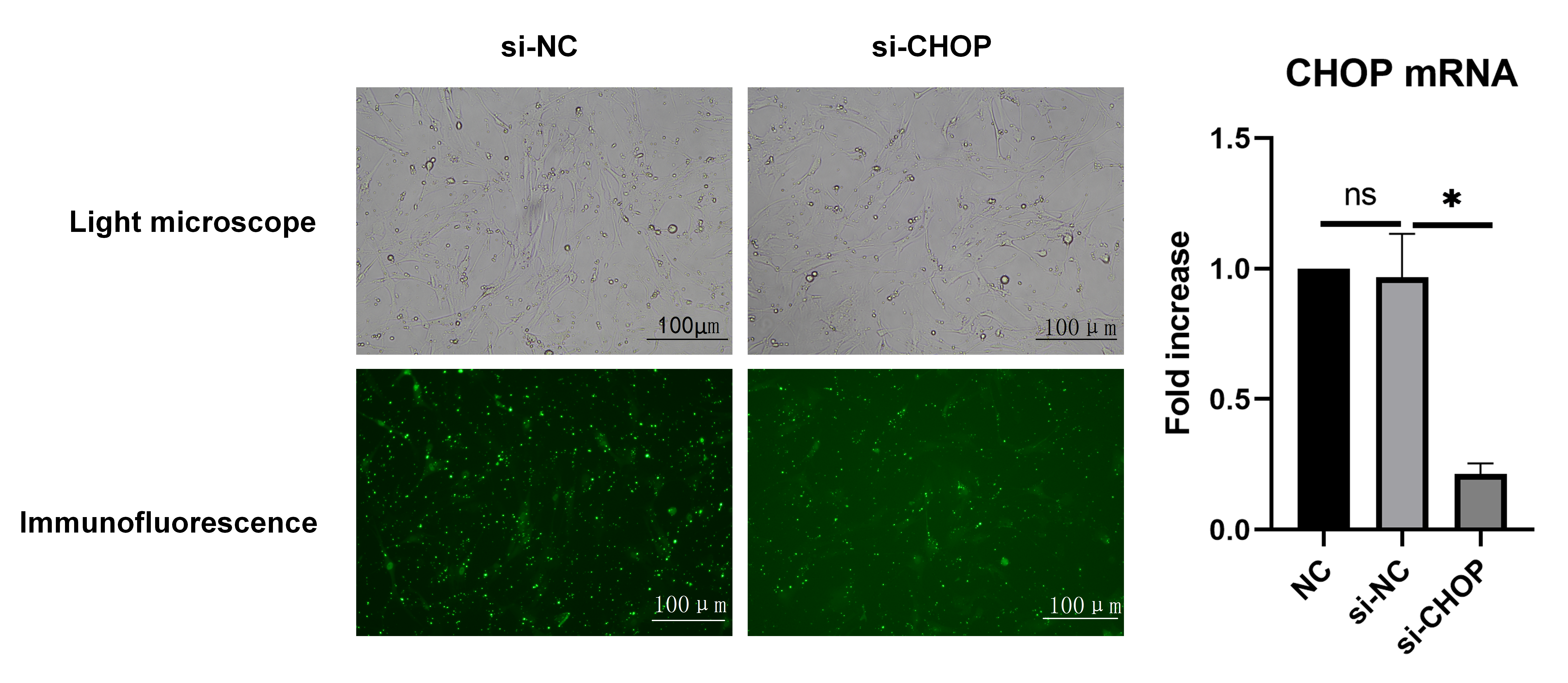

Supplement: Supplemental Material [file IRNF_A_2027248_SM9104.png]
